# Atypical Antibody Dynamics During Human Coronavirus HKU1 Infections

**DOI:** 10.3389/fmicb.2022.853410

**Published:** 2022-04-27

**Authors:** Ferdyansyah Sechan, Marloes Grobben, Arthur W. D. Edridge, Maarten F. Jebbink, Katherine Loens, Margareta Ieven, Herman Goossens, Susan van Hemert-Glaubitz, Marit J. van Gils, Lia van der Hoek

**Affiliations:** ^1^Laboratory of Experimental Virology, Department of Medical Microbiology and Infection Prevention, Amsterdam UMC, University of Amsterdam, Amsterdam, Netherlands; ^2^Amsterdam Institute for Infection and Immunity, Amsterdam, Netherlands; ^3^Department of Medical Microbiology, Vaccine & Infectious Disease Institute (VAXINFECTIO), University of Antwerp, Antwerpen, Belgium; ^4^Department of Microbiology, University Hospital Antwerp, Edegem, Belgium; ^5^Julius Centre for Health Sciences and Primary Care, University Medical Centre Utrecht, Utrecht University, Utrecht, Netherlands

**Keywords:** endemic seasonal coronavirus, HCoV-HKU1, HCoV-OC43, HCoV-229E, HCoV-NL63, nucleocapsid protein, spike protein, IgG response

## Abstract

Human coronavirus HKU1 (HCoV-HKU1) is one of the four endemic coronaviruses. It has been suggested that there is a difference in incidence, with PCR-confirmed HCoV-NL63 and HCoV-OC43 infections occurring more commonly, whereas HCoV-HKU1 is the least seen. Lower incidence of HCoV-HKU1 infection has also been observed in serological studies. The current study aimed to investigate antibody dynamics during PCR-confirmed HCoV-HKU1 infections using serum collected during infection and 1 month later. We expressed a new HCoV-HKU1 antigen consisting of both the linker and carboxy-terminal domain of the viral nucleocapsid protein and implemented it in ELISA. We also applied a spike-based Luminex assay on serum samples from PCR-confirmed infections by the four endemic HCoVs. At least half of HCoV-HKU1-infected subjects consistently showed no antibody rise *via* either assay, and some subjects even exhibited substantial antibody decline. Investigation of self-reported symptoms revealed that HCoV-HKU1-infected subjects rated their illness milder than subjects infected by other HCoVs. In conclusion, HCoV-HKU1 infections reported in this study displayed atypical antibody dynamics and milder symptoms when compared to the other endemic HCoVs.

## Introduction

Human coronavirus (HCoV) HKU1 is a positive-strand RNA virus that belongs to the genus *Betacoronavirus*. In the year 2005, this virus was reported for the first time in a 71-year-old male patient for the first time with unexplainable pneumonia (Woo et al., 2005). Together with the other endemic HCoVs (HCoV-229E, HCoV-OC43, and HCoV-NL63), it is known as a common cold coronavirus infecting humans. The virus has a worldwide distribution (Vabret et al., 2008; Mackay et al., 2012; Liu et al., 2014; Al-Khannaq et al., 2016; Yip et al., 2016; Killerby et al., 2018; Masse et al., 2020). There are at least three co-circulating genotypes of HCoV-HKU1: A, B, and C, with recombination occurring frequently among them (Woo et al., 2006). Studying the HCoV-HKU1 infections in population remains challenging, because this virus is the least frequently detected HCoV. For example, the RT-PCR test to diagnose HCoV-HKU1 was discontinued in Scotland, in 2012, due to low detection rate (Nickbakhsh et al., 2020).

The four endemic HCoVs are suspected of having animal origins (Cui et al., 2019), similar to SARS-CoV-2. However, unlike SARS-CoV-2, they have circulated in humans for centuries, and infection mostly results in non-life-threatening common-cold-like symptoms. Considering that SARS-CoV-2 may soon become an endemic human coronavirus, possibly sharing epidemic and/or pathogenic characteristics with other endemic HCoVs, we were interested in the features that these four viruses share, and also those that separate them. The HCoVs share the characteristic that first infections occur in early childhood (Dijkman et al., 2012; Zhou et al., 2013), protective immunity is short-lived, and reinfection in humans occurs frequently (Edridge et al., 2020; Galanti and Shaman, 2020; Petrie et al., 2021). Serological studies show that HCoV-HKU1 (re-)infection occurs the least comparatively (Dijkman et al., 2012; Edridge et al., 2020). Edridge et al also showed that some HCoV-HKU1 infections are not accompanied by increased antibody titer (Edridge et al., 2020). In that particular study, a partial nucleocapsid protein (N) of HCoV-HKU1 was used as the respective ELISA antigen.

The N protein of coronaviruses is structural, and the tertiary structure is conserved across different coronavirus species. There are two structural domains: the C-terminal (Ct) and N-terminal (Nt) domains, connected by a flexible linker domain (McBride et al., 2014; Zhou et al., 2020). Due to the high amino acid sequence identity, the use of full N antigen in serology can result in cross-reactivity between HCoV species belonging to the same genus (Lehmann et al., 2008). The Ct domain of N (NCt) is used in many serology studies since *Alphacoronavirus* serology was identified to be most specific (Mourez et al., 2007; Dijkman et al., 2008; Severance et al., 2008; Sastre et al., 2011). However, it could be that the NCt antigen of HCoV-HKU1 contains too few epitopes, thus, explaining the lack of HKU1-antibody increase (Edridge et al., 2020). The HKU1-antibody ELISA could potentially be improved by expressing and using an HCoV-HKU1 antigen that includes the linker (L) domain as well as the NCt domain (NLCt protein). The flexible linker domain of HCoV-HKU1 N-protein is most probably immunogenic since the linker domain of HCoV-OC43 N-protein also displays immunogenicity (Liang et al., 2013). Furthermore, since recent studies of SARS-CoV-2 infection revealed that spike (S) and N antibodies might have different dynamics, we anticipated that a serological assay using S protein may also improve the HKU1-serology (Brochot et al., 2020; Röltgen et al., 2020). Thus, we included S-antibody Luminex tests for all four HCoVs in the current study. The study aimed to determine whether HCoV-HKU1 antibody dynamics truly differ from those of other endemic human coronaviruses.

## Materials and Methods

### Serum Samples

A total of 108 serum samples were obtained from 54 subjects with a PCR-confirmed endemic HCoV infection. The number of subjects with HCoV-HKU1, HCoV-OC43, HCoV-NL63, and HCoV-229E was 13, 14, 11, and 16, respectively. The causative agent of disease for each subject was confirmed using multiplex RT-PCR assay on the nasopharyngeal sample (Loens et al., 2012). A cycle threshold (Ct) value of 30 and lower was used as a cutoff to select subjects with confirmed HCoV infection, except for HCoV-HKU1. For HCoV-HKU1, the RespiFinder test was used (PathoFinder, Maastricht, The Netherlands). The Respifinder assay, version RespiFinder plus - RespiFinder Smart 21, allows testing specifically for HCoV-HKU1, yet no cycle threshold value could be derived.

The 54 subjects were part of the Genomics to combat Resistance against Antibiotics in Community-acquired LRTI in Europe (GRACE) study. Participants were recruited between November 2007 and April 2010 by primary care practitioners in 16 networks from 12 European countries. The details of the GRACE cohort study are described in detail elsewhere (Ieven et al., 2018). Subjects aged 18 years or above with acute or worsened cough, or other symptoms indicating lower respiratory tract infection (LRTI), as reported by their general practitioner (GP), were invited to participate, and written consent was obtained before asking for participation. All recruiting GPs received standardized sampling material and a protocol with detailed instructions on the sampling of the patients. Within 24 h of first presentation and inclusion, blood, sputum if available, and two nasopharyngeal flocked swabs (NPS) (Copan Italia, Brescia, Italy) were taken [visit 1 (V1)]. At days 28–35, serum sampling and the two NPS were repeated [visit 2(V2)]. Serum, and NPS were stored frozen in the local laboratories until regular shipment to the central laboratory (University Hospital Antwerp), where specimens were stored at -80°C until analysis.

Demographic data and clinical manifestation of LRTI for each subject were recorded using a standardized case report form (CRF) that was completed by the GP during V1. Demographic data included age, gender, presence of comorbidities, and the estimated duration of illness or cough before the first visit. Within the CRF, subjects were also asked whether any of the 14 symptoms/conditions were present, and to rate the severity in case any symptom was present. These 14 symptoms are cough, phlegm, shortness of breath, wheezing, runny nose, fever, chest pain, muscle ache, headache, disturbed sleep, generally feeling unwell, interference with normal daily activities, confusion/disorientation, and diarrhea. The severity of each symptom was scored as follows: 0 = symptom absent/not a problem, 1 = mild problem, 2 = moderate problem, 3 = severe problem, and mean symptom severity score (SSS) for each subject was calculated as average score value from all 14 symptoms (Vos et al., 2021). Clinical data of one subject infected with HCoV-NL63 and three subjects infected with HCoV-229E were missing, therefore, 50 subjects were included in the demographic and symptom analyses.

The GRACE study was approved by the local ethics committees in all participating centers and by the competent authorities in each country: Cardiff and Southampton (United Kingdom): Southampton and South West Hampshire Research Ethics Committee A; Utrecht (Netherlands): Medisch Etische Toetsingcommissie Universitair Medisch Centrum Utrecht; Barcelona (Spain): Comité étic d’Investigació clínica Hospital Clínic de Barcelona; Mataro (Spain): Comitè d’Ètica d’Investigació Clínica (CEIC) del Consirci Sanitari del Maresme; Rotenburg (Germany): Ethik-Komission der Medizinischen Fakultät der Georg-August-Universitat Göttingen; Antwerp (Belgium): UZ Antwerpen Comité voor Medische Ethiek; Lodz, Szeczecin and Bialystok (Poland): Komisja Bioetyki Uniwersytetu Medycznego W Lodzi; Milano (Italy):IRCCS Fondazione Cà Granda Policlinico; Jonkoping (Sweden): Regionala etikprövningsnämden I Linköping; Bratislava (Slovakia): Etika Komisia Bratislavskeho; Gent (Belgium): Ethisch Comité Universitair Ziekenhuis Gent; Nice (France): Comité de Protection des Personnes Sud-Méditerranée II, Hôpital Salvator; and Jesenice (Slovenia): Komisija Republike Slovenije za Medicinsko Etiko.

### Design of Recombinant Coronavirus HKU1 Nucleocapsid Protein

**FIGURE 1:**
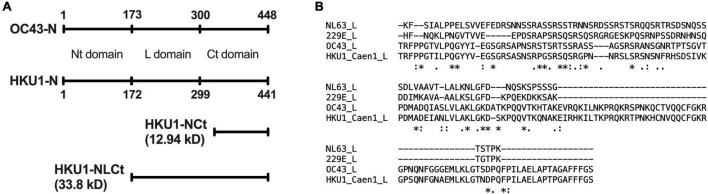
**(A)** Location of Nt-, Ct-, and linker (L)-domain of HCoV-HKU1 N as determined by pairwise alignment with HCoV-OC43, as well as the comparison of size between HKU1-NLCt and the HKU1-NCt antigen. **(B)** Multiple alignment of the nucleocapsid L domain of HCoV-NL63, HCoV-229E, HCoV-OC43, and HCoV-HKU1. The symbols below the aligned amino acid sequence represent the consensus symbol of amino acid residue at each position: asterisks (*) denotes fully conserved residue, colons (:) denotes amino acid residues with strongly similar property, periods (.) denotes amino acid residues with weakly similar property, and the absence of symbol denotes completely different property.

The location of HCoV-OC43 linker domain (Liang et al., 2013) was aligned with the amino acid sequence of HCoV-HKU1 N using global pairwise alignment (Madeira et al., 2019). GenBank accession number AAR01019.1 (HCoV-OC43 isolate VR-759) and ADN03343.1 (HCoV-HKU1 isolate Caen1) were used for the alignment. It was estimated that the linker domain of HCoV-HKU1 N was located between amino acids 172 and 299. Therefore, the new antigen “NLCt” was designed to start at amino acid 172 and run to the end (amino acid 441) (Figure 1A). Multiple sequence alignment was done between HCoV-HKU1 NLCt amino acid sequence with corresponding sequences on other endemic coronaviruses to estimate the possibility of cross-reaction between species. HKU1-NLCt shared 64.33% percent identity with HCoV-OC43 while the values are lower for HCoV-NL63 and HCoV-229E (percent identity of 24.56 and 24.85%, respectively). The presumed L domain of HCoV-NL63 and HCoV-229E (Figure 1B) was also found by amino acid alignment using HCoV-OC43 N protein as the reference (Liang et al., 2013). Furthermore, to account for the possibility that antibodies against one genotype could not recognize epitopes from other genotypes, the amino acid sequence of the NLCt protein from one representative of each genotype as mentioned by Woo et al. (2006) were aligned with the NLCt sequence based on HCoV-HKU1 strain Caen1. The sequence shared is used to construct a phylogenetic tree alongside the Caen1 nucleocapsid sequence. The Caen1 strain clustered with HCoV-HKU1 genotype A. The HKU1-NLCt antigen we produced shared > 94% identity with the corresponding sequence from HCoV-HKU1 genotypes B and C.

### Expression of Recombinant Coronavirus HKU1 Nucleocapsid Protein

The production of the HKU1-NLCt antigen, starting with the generation of the plasmid construct to the expression of the protein, was done similarly to previously described methods (Dijkman et al., 2012). In short, the HKU1-NLCt gene fragment was amplified using PCR with Q5 High Fidelity DNA Polymerase (New England Biolabs, Ipswitch, MA, United States). The template used in the PCR was the full nucleocapsid protein gene from HCoV-HKU1 strain Caen1 (GenBank accession number HM0384837.1), and the sequences for the forward and reverse primer were 5′ – CACCACTAGGTTTCCGCCTG – 3′ (5′-HKU1-NLCt) and 5′–TTAAGCAACAGAGTCTTCTACATAAG–3′ (3′-HKU1-end), respectively. The PCR product was cloned into pET/100/D-TOPO expression plasmids (Thermo Fisher Scientific, Waltham, MA, United States), which were transformed into *Escherichia coli* DH5α competent cells (Thermo Fisher Scientific). Sequencing on the generated pET-HKU1-NLCt plasmid confirmed that the cloned gene was identical to Caen1. The HKU1-NLCt antigen was then expressed and purified using steps described previously (Dijkman et al., 2008). Since the first purified product still contained impurities (assumed to be bacterial proteins, Supplementary Figure 1A), the purification step was done two times (Supplementary Figure 1B).

### HKU1 Nucleocapsid ELISA

The NLCt ELISA protocol used in this study was similar to the one previously described (Edridge et al., 2020), with some modifications. The antigen was coated overnight on 96-well half-area microplates (Greiner Bio-One, Alphen aan de Rijn, The Netherlands), with 3 μg/ml antigen in 0.1 M carbonate buffer, at pH 9.6. The plates were incubated for 1 h at 20*^o^*C with 5% skimmed milk (Honeywell Fluka, Landsmeer, The Netherlands) solution in PBS with 0.1% Tween (PBST) to block non-specific binding sites, followed by three washing steps with PBST. The serum samples were diluted at 1:200 in PBST supplemented with 1% skimmed milk. The diluted serum samples were added to the washed plates in duplicate and incubated for 1 h at 20°C, followed by a washing step. After that, alkaline phosphatase-conjugated AffiniPure Goat Anti-Human IgG, Fc Fragment Specific (Jackson ImmunoResearch, West Grove, PA, United States), diluted 1:1,500 in 1% skimmed milk and PBST solution was added. Plates were incubated for 1 h at 20°C, followed by washing. Finally, the luminescence signal was developed using LumiPhos (Lumigen, Southfield, MI, United States) and the plates were incubated in the dark for 20 min at 20°C. The luminescence (ELISA signal value) was measured using GloMax 96 Microplate Luminometer (Promega, Madison, WI, United States). The final ELISA signal value for each serum sample was calculated as the average of the two measurements. ELISA signal fold-change was calculated by dividing the value of ELISA signal from V2 with the value from V1.

A cutoff value of 1.40 was used for significant antibody rise in response to an infection. This cutoff was established previously by Edridge et al. (2020), by measuring the natural fluctuation in measles virus antibodies in consecutive samples from 10 individuals, assuming that measles infection did not occur during follow-up. Fold changes in ELISA signal for measles antibodies ranged between 0.85 and 1.28. Edridge et al. subsequently showed that for the coronaviruses, outliers were found for signal fold changes ≥1.40 by evaluating the distribution of the signal fold change for each of the seasonal coronaviruses. In addition, Edridge et al confirmed that self-reported influenza-like illnesses directly preceded the ≥1.40-fold rise in antibodies. Finally, the ELISA ≥1.40 rise of HCoV-NL63 were compared with neutralization titers for HCoV-NL63 and an increase in neutralization titer indeed accompanied a ≥1.40-fold rise in antibodies.

### Recombinant Prefusion Spike Coronavirus Luminex Assay

The Luminex assay using prefusion S antigen of endemic HCoVs was done as previously described (Grobben et al., 2021). For HCoV-HKU1, the S antigens were stabilized in the prefusion conformation by mutating two amino acid residues at position 1067 and 1068 into two prolines and by substitution of the furin cleavage site with amino acid sequence GGSGS to prevent cleavage and subunit dissociation during the production process (Kirchdoerfer et al., 2016; Brouwer et al., 2021). The S antigen of HCoV-HKU1 was derived from isolate N5 (NCBI accession code: Q0ZME7), which belongs to genotype C (Woo et al., 2006). The protein was expressed in HEK293F cells (Invitrogen, Waltham, MA, United States) in a Freestyle medium and purified with affinity chromatography using NiNTA agarose beads (Qiagen, Venlo, The Netherlands). Each antigen was then coupled to Luminex Magplex beads using a two-step carbodiimide reaction as previously described (Grobben et al., 2021; Keuning et al., 2021). The coupled beads (20 beads per μl of each S of endemic HCoVs), together with serum samples diluted to 1:10,000, were used in the Luminex assay, with Goat-anti-human IgG-PE (SouthernBiotech, Birmingham, AL, United States) as the secondary antibody. Positive and negative controls (tetanus toxoid-coupled beads and uncoupled beads, respectively) were included in each run. The readout was expressed as Median Fluorescence Intensity of at least 50 beads per antigen. Fold change values were calculated as the ratio of V2:V1. The cutoff for significant antibody rise or antibody decrease was defined as the upper fence (Q3 + 1.5*IQR) or lower fence (Q1 – 1.5*IQR) of fold change values derived from tetanus toxoid-coupled beads (positive control) (Supplementary Figure 2). The cutoff values were defined as 2.01 and 0.23 for antibody rise and antibody decrease, respectively.

### Statistical Analysis

Statistical analyses were done using SPSS version 27 (IBM, Armonk, NY, United States) and R version 4.0.3, while the figures were made with Prism version 9.3.0 (GraphPad, San Diego, CA, United States). Correlations between two (non-parametric) numerical variables were determined using Spearman’s Rank Order Correlation. The distribution of categorical variables between groups was compared using Fisher’s exact test. Numerical variables between HCoV-HKU1 subjects and other HCoV subjects (pooled into one group) were compared for the difference using the Mann–Whitney *U* test. The effect of HCoV-HKU1 infection on the possible influence of age as a confounding factor for selecting “illness interfering with daily activities” (binomial categorical variable, yes-no) as “no” was investigated using logistic regression.

## Results

**FIGURE 2:**
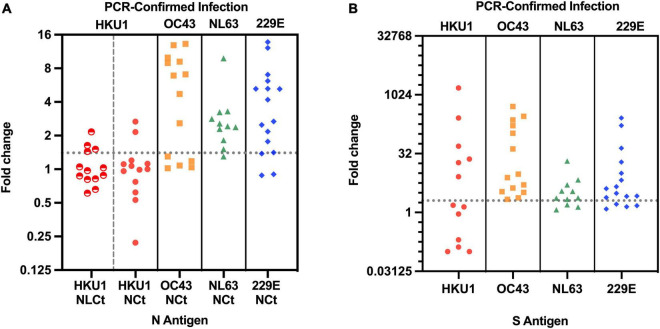
**(A)** V1-V2 Fold change in antibody values of 54 subjects with PCR-confirmed endemic HCoV infection, as assayed with ELISA with matched N antigen. For HCoV-HKU1-infections, both HKU1-NLCt (half-filled red circles) and HKU1-NCt (full red circles) were used for the N assay. Dotted line represents cutoff of significant antibody rise (fold change of 1.40 or higher). The NCt ELISA fold change values for all four endemic coronaviruses have been published previously Edridge et al. (2020). **(B)** V1-V2 Fold change in antibody values using matched S antigens for each HCoV infection in the S-Luminex assay. Dotted line represents the cutoff of significant S-antibody rise (fold change of 2.01 or higher).

A total of 13 subjects with lower respiratory tract infection that visited their GP with a PCR-confirmed HCoV-HKU1 infection were included in the study. We tested for antibodies in serum collected at two time points. The first time point (V1) was the moment of the GP visit, which is also the date of PCR-positive testing for HCoV-HKU1. The second serum sampling moment (V2) was 1 month later, at that time the patient had recovered and the PCR test for HCoV-HKU1 was negative. We expressed a protein containing the Ct part and the linker domain of the HCoV-HKU1 N-protein in *E. coli*, to be used as antigen in HKU1-ELISA antibody tests (Figure 1A and Supplementary Figure 1). V1 and V2 serums were tested using ELISA and the rise or decrease was measured *via* fold change. A V1-V2 fold change of >1.40 represents a significant rise (Edridge et al., 2020). Four of 13 (31%) subjects were presented with a significant V1-V2 NLCt-antibody ELISA signal increase (Figure 2A). To investigate whether this low frequency of HKU1-antibody response only occurs for antibodies recognizing the N-protein, we also tested for antibodies recognizing the S protein using the HKU1-S Luminex assay. Six of the 13 (46%) subjects showed a significant S-antibody rise (Figure 2B). Curiously, of the other seven samples, more than half (57%, 4 of 7) exhibited a significant decrease in antibodies (fold change values of 0.20 or lower, Figure 2B and Table 1, values in red). In two of these subjects, HK-01 and HK-11, the fold change values by both HKU1-NLCt and HKU1-NCt antigens were significantly decreased (<0.7) while the other two subjects (HK-06 and HK-07) showed fold change values close to 1 for HKU1-NLCt and HKU1-NCt. One subject (HK-09) showed a decrease with a fold change of 0.22 by HKU1-NLCt antigen ELISA, but the S or NLCt fold change values showed no significant difference (fold change of 1,39 and 0,81, respectively).

**TABLE 1:** The V1-V2 antibody fold change values of individual HCoV-HKU1-infected-subjects assayed by HKU1 S, NLCt, and NLCt antigen.

HKU1 subject	HKU1 antigen fold change		
	S	NLCt	NCt
HK-01*^c^*	**0,13**	**0,66**	**0,53**
HK-02	0,91	1,03	1,07
HK-03*^a^*	**49,57**	**2,16**	**2,67**
HK-04*^b^*	261,57	1,05	0,99
HK-05	1,53	0,97	0,96
HK-06*^d^*	0,10	0,82	0,77
HK-07*^d^*	0,10	0,88	1,00
HK-08*^b^*	18,82	0,87	1,12
HK-09	1,39	0,81	0,22
HK-10*^a^*	**1534,43**	**1,51**	1,20
HK-11*^c^*	0,20	0,61	0,62
HK-12*^a^*	**8,22**	**1,44**	1,11
HK-13*^a^*	**23,18**	**1,63**	**2,15**

*^a^Subject with fold change by S assay and either of the N assays above the cutoff value (fold change value in black and bold).*

*^b^Subject with only fold change by S assay above the cutoff value (fold change value in black and underlined).*

*^c^Subject with fold change by S assay and either of the N assays below the cutoff value (fold change value in red and bold).*

*^d^Subject with only fold change by S assay below the cutoff value (fold change value in red and underlined).*

We investigated 41 subjects infected by HCoVs other than HCoV-HKU1 (HCoV-NL63 *n* = 11; HCoV-OC43 *n* = 14; HCoV-229E *n* = 16). In 80% (33 of 41) of these subjects, ELISAs using respective NCt antigen showed antibody rises above the cutoff (Figure 2A), and the same was observed in the fold change by the respective S Luminex assay, with significant antibody increasein 35 out of the 41 subjects (Figure 2B). In total, the difference of antibody rise/no rise between HCoV-HKU1 and the other HCoVs was significantly distinct for both assays (S-Luminex Fisher’s exact test *p* = 0.025; N-ELISA Fisher’s exact test, *p* = 0.002). Furthermore, none of the NL63, OC43, or 229E-infected subjects presented with significant antibody decreases between V1 and V2.

We subsequently examined whether patient characteristics for the HCoV-HKU1-infected subjects were different compared to the subjects infected by other coronaviruses. The demographic data, including age, gender, ethnicity, smoking history, possible comorbidities (8 items), symptom manifestation at the beginning of the disease (14 items), and estimated duration of prior illness and prior cough, were compared and summarized in Table 2 for each endemic HCoV. The HKU1-infected subjects tended to be younger than other subjects (median age 38 years for HKU1-infected subjects *versus* 53 years for other subjects; Mann–Whitney *U* test, *p* = 0.029), but no difference was observed for other demographic characteristics. The vast majority of our study subjects were white, and the possible influence of racial background could thus not be measured in the study.

**TABLE 2:** Demographic data comparison between subjects with infection by endemic HCoVs.

Demographic^a^	HKU1 *n* = 13	OC43 *n* = 14	NL63 *n* = 10	229E *n* = 13
Age	**38 (26–56)^e^**	55 (29–74)	46.5 (22–64)	53 (21–75)
Male gender	4 (30.8)	5 (35.7)	4 (40)	6 (46.2)
White racial background	12 (92.3)	14 (100)	10 (100)	13 (100)
Smoking past/present	8 (61.5)	7 (50)	2 (20)	4 (30.8)
**Comorbidity**				
COPD	0	1 (7.1)	0	2 (15.4)
Asthma	0	1 (7.1)	0	2 (15.4)
Other lung disease	0	0	**2 (20)^c^**	0
Heart failure	0	0	0	0
Ischemic heart disease	0	0	0	1 (7.7)
Other heart disease	1 (7.7)	1 (7.1)	0	1 (7.7)
Diabetes	0	0	1 (10)	0
Prev. hospitalization	1 (7.7)	0	0	0
**Symptoms**				
Cough	13 (100)	14 (100)	10 (100)	13 (100)
Phlegm	12 (92.3)	12 (85.7)	7 (70)	9 (69.2)
Shortness of breath	6 (46.2)	9 (64.3)	5 (50)	7 (53.8)
Wheezing	4 (30.8)	7 (50)	3 (30)	2 (15.4)
Runny nose	10 (76.9)	13 (92.9)	7 (70)	11 (84.6)
Fever	3 (23.1)	7 (50)	6 (60)	2 (15.4)
Chest pain	3 (23.1)	6 (42.9)	5 (50)	7 (53.8)
Muscle ache	5 (38.5)	8 (57.1)	4 (40)	5 (38.5)
Headache	9 (69.2)	9 (64.3)	7 (70)	10 (76.9)
Disturbed sleep	5 (38.5)	8 (57.1)	8 (80)	8 (61.5)
Feeling generally unwell	11 (84.6)	13 (92.9)	8 (80)	11 (84.6)
Interference of daily activity	**6 (46.2)^c^**	13 (92.9)	9 (90)	11 (84.6)
Confusion/disorientation	0	3 (21.4)	0	1 (7.7)
Diarrhea	0	**4 (28.6)^d^**	0	0
Mean SSS^b^	**0.6 (0.4–1.7)^e^**	1.1 (0.5–2.1)	1.3 (0.4–2.4)	0.9 (0.3–1.4)
Duration of prior illness	7 (1–21)	5 (2–14)	3 (1–20)	4 (2–20)
Duration of prior cough	**8 (1–28)^e^**	5 (2–20)	3 (1–20)	4 (1–20)

*^a^Data is presented as either median (age, mean symptom severity score, duration of prior illness, and duration or prior cough, with range between brackets), or frequency (gender, Caucasian ethnicity, presence of comorbidity, and symptom presentation, with percentage between brackets).*

*^b^Mean symptom score is the average of symptom score for each of the 14 symptoms (0 = no problem, 1 = mild problem, 2 = moderate problem, 3 = severe problem) (Vos et al., 2021). No cutoff of severity was established for this score.*

*^c^In bold: significance by Fisher’s exact test, p < 0.05.*

*^d^In bold: Significance by Fisher’s exact test, p < 0.001.*

*^e^In bold: Significance by Mann-Whitney U test, p < 0.05.*

The HCoV-HKU1 infection was associated with a lower risk of interference with normal daily activities (including work/study, housework, family, and leisure activities, odds ratio (OR) 0.10, 95% confidence interval (CI) 0.02–0.45, *p* = 0.003). When we corrected for age—given HKU1-infected individuals were slightly younger—a similar association was found (OR 0.04, 95% CI 0.00–0.26, *p* = 0.002). The subjectively milder symptoms of HCoV-HKU1 infection were also reflected by a significantly lower mean symptom severity score compared to other subjects (median value of 0.6 and 1.1 for HCoV-HKU1-infected subjects and other subjects, respectively, Mann–Whitney *U* test, *p* = 0.025). Furthermore, we noticed that more people in the OC43-infected group had diarrhea (Fisher’s exact test, *p* < 0.001).

When we looked at all subjects we observed that the symptom severity score was negatively associated with duration of prior illness (Spearman’s rho = –0.385, *p* = 0.006) and also with the duration of prior cough (Spearman’s rho = –0.298, *p* = 0.004). In theory, it could mean that HCoV-HKU1 infected people experienced less interference with daily activity, and therefore waited longer before seeking medical care. If this was the case, the peak in antibody levels could have been close to the date of V1 serum collection. Indeed, a high S-antibody level at V1 was found in three of the four people with a steep decrease in antibodies. This was less visible for the antibodies recognizing the NLCt or NCt (Supplementary Figures 3, 4 and Supplementary Tables 1, 2). To test this hypothesis further, we investigated for all HCoVs whether the duration of illness or cough prior to enrollment was associated with antibody dynamics at V2. Across all subjects, we found a significant negative correlation between the duration of prior illness before visiting the GP and the fold change value using the S-Luminex assay as readout (Spearman’s rho = –0.280, *p* = 0.049). Although the number of subjects was low, we also examined three categories of the subject within the HCoV-HKU1 infections (V1–V2 antibody rise, antibody decrease, or stable antibody levels), but observed no significant link between any demographic category, or the duration of disease prior to the GP visit, and HKU1-antibody dynamics (Table 3).

**TABLE 3:** Association between demographic categories and S-antibody dynamics in HCoV-HKU1-infected symptoms.

Demographic categories (13)	S antibody stable (3)	S antibody rise (6)		S antibody fall (4)	
	N	N (%) ^a^	*p*-value	N (%) ^a^	*p*-value
Age 41 or over (6)	1	2 (33)	0.592	3 (75)	1
Male (4)	2	1 (17)	0.559	1 (25)	1
Presence of comorbidity (2)	1	0	0.462	1 (25)	1
Smoking past/present (8)	1	5 (83)	0.266	2 (50)	1
Prior illness ≥ 1 week (8)	2	2 (33)	0.103	4 (100)	0.105
Prior cough ≥ 1 week (9)	2	4 (67)	1	3 (75)	1
Phlegm (12)	3	5 (83)	0.462	4 (100)	1
Shortness of breath (6)	2	2 (33)	0.592	2 (50)	1
Wheezing (4)	1	0	0.070	3 (75)	0.217
Runny nose (10)	2	6 (100)	0.192	2 (50)	0.203
Fever (3)	1	1 (17)	1	1 (25)	1
Chest pain (3)	1	0	0.192	2 (50)	0.203
Muscle ache (5)	1	2 (33)	1	2 (50)	1
Headache (9)	3	4 (67)	1	2 (50)	0.537
Disturbed sleep (5)	2	1 (17)	0.266	3 (75)	1
Feeling generally unwell (11)	2	6 (100)	0.462	3 (75)	1
Interference of daily activities (6)	2	3 (50)	1	1 (25)	0.266
Mean SSS is average^b^ or higher (6)	0	3 (50)	1	3 (75)	0.267

*^a^To compare the proportion between antibody rise and non-rise, subjects with antibody fall and antibody stable were grouped as non-rise. Similarly, to compare the proportion between fall and non-fall, subjects with antibody rise and antibody stability were coded as non-fall. Fisher’s exact test was used to calculate p-value between antibody rise vs. no-rise, as well as between antibody fall vs. no-fall.*

*^b^The average of a mean symptom score for people with HCoV-HKU1 infection is 0.7 (Table 2).*

## Discussion

Here we report that a substantial portion of people infected by HCoV-HKU1 displays no rise in antibodies following infection. This unusual phenomenon makes HCoV-HKU1 noteworthy, as infections due to HCoV-OC43, HCoV-NL63, and HCoV-229E result in more typical antibody dynamics. We hypothesize that this difference is a result of lower disease severity in HCoV-HKU1 infection. We observed a less pronounced impact on daily life experienced by our HCoV-HKU1-infected subjects. A correlation between a rise in antibodies and disease severity has not been studied for the endemic HCoVs but has been reported for SARS-CoV-2. Higher neutralizing antibody titers were observed in people with more severe disease in comparison to mild cases (Brochot et al., 2020; Ren et al., 2020; Röltgen et al., 2020; Legros et al., 2021). Additionally, the presence of antibodies recognizing N protein is associated with more severe COVID-19 (Sen et al., 2021). The lack of increased antibody titer observed in half of our patients infected with HCoV-HKU1 may thus reflect the mild nature of many HCoV-HKU1 infections.

We also identified cases that showed a significant decline in the HKU1-IgG antibodies. The extended time between the onset of symptoms and GP visits observed for HCoV-HKU1 infected subjects may play a role here. In addition, it could be that HCoV-HKU1 viruses capture virus-specific HKU1-IgGs. This, in combination with a situation where HKU1-specific IgGs are not newly produced, may lead to a decreasing level of HCoV-HKU1 recognizing IgGs. In this situation, it could theoretically be that infection-induced production of secretory IgAs, instead of IgGs, is playing a role in clearing the infection (Callow, 1985; Habibi et al., 2015). Indeed, Gorse et al. found that the prevalence in which secretory IgA was found in the nasal washing of adults with seasonal coronavirus infections were higher for HCoV-HKU1 (31%) than HCoV-OC43, HCoV-229E, and HCoV-NL63 (22, 11, and 8%, respectively) (Gorse et al., 2010). Future research may be considered for testing HKU1-recognizing sIgA in nasopharyngeal samples of HCoV-HKU1-infected subjects, therefore, investigating whether higher sIgA levels are indeed found in people who display no HKU1-specific IgG rise.

By using HKU1-NLCt in our ELISA, we found that two times as many subjects had antibody fold change values above the cutoff, when compared with the previously used HKU1-NCt antigen (Dijkman et al., 2012; Edridge et al., 2020). This indicates that a limited antigenicity may play a role when NCt is used. At the same time, we did not find substantially more cross-reactivity by antibodies induced by HCoV-OC43 infections (data not shown). Since the HKU1-NLCt antigen contains one additional domain, it is tempting to suggest that using the whole N of HCoV-HKU1 may further improve the assay’s sensitivity. However, due to its conserved structure, the use of whole HCoV-HKU1 N in ELISA may result in considerable cross-reactivity between HCoV-HKU1 and HCoV-OC43, as has been previously reported (Lehmann et al., 2008).

Our study does have some weaknesses. First, the PCR assay designs for HCoVs were not identical. The HCoV-HKU1 infections were identified using a commercial molecular assay that provided no information on the virus load, whereas the other HCoVs were diagnosed *via* quantitative PCRs developed in-house (Loens et al., 2012). The NL63-, OC43-, and 229E-infections all scored Ct values below 30; however, we could not identify the virus concentration in the nasopharyngeal swabs from HCoV-HKU1 infections. Secondly, we were not able to determine the HKU1 genotypes infecting our subjects. There are at least three co-circulating genotypes of HCoV-HKU1: genotypes A, B, and C (Woo et al., 2006), and it is possible that the genotypes of HCoV-HKU1 in our cohort did not match with the genotype used for the ELISA test. However, we observed similar findings for both S and N antigens, and these antigens were derived from different HCoV-HKU1 genotypes, with the NCt and NLCt antigens expressed from isolate Caen1 (genotype A), and the S antigen expressed from isolate N5 (genotype C). Furthermore, similar to HCoV-HKU1, HCoV-OC43 and HCoV-NL63 also have several co-circulating genotypes. If a mismatch between ELISA antigen and infecting genotype would result in false-negative responses, this is likely to have also occurred for HCoV-OC43 and HCoV-NL63.

In conclusion, we demonstrate that an HKU1-specific-IgG rise is a poor marker of an HCoV-HKU1 infection. These findings contribute to explaining the low detectability of HCoV-HKU1 infections by serology, and we may consider HCoV-HKU1 to be an interesting atypical endemic coronavirus.

## Data Availability Statement

The original contributions presented in the study are included in the article/Supplementary Material, further inquiries can be directed to the corresponding author.

## Ethics Statement

The studies involving human participants were reviewed and approved by Cardiff and Southampton (United Kingdom): Southampton & South West Hampshire Research Ethics Committee A; Utrecht (Netherlands): Medisch Etische Toetsingcommissie Universitair Medisch Centrum Utrecht; Barcelona (Spain): Comité Étic d’Investigació Clínica Hospital Clínic de Barcelona; Mataro (Spain): Comitè d’Ètica d’Investigació Clínica (CEIC) del Consirci Sanitari del Maresme; Rotenburg (Germany): Ethik-Komission der Medizinischen Fakultät der Georg-August-Universitat Göttingen; Antwerp (Belgium): UZ Antwerpen Comité voor Medische Ethiek; Lodz, Szeczecin and Bialystok (Poland): Komisja Bioetyki Uniwersytetu Medycznego W Lodzi; Milano (Italy):IRCCS Fondazione Cà Granda Policlinico; Jonkoping (Sweden): Regionala etikprövningsnämden I Linköping; Bratislava (Slovakia): Etika Komisia Bratislavskeho; Gent (Belgium): Ethisch Comité Universitair Ziekenhuis Gent; Nice (France): Comité de Protection des Personnes Sud-Méditerranée II, Hôpital Salvator; and Jesenice (Slovenia): Komisija Republike Slovenije za Medicinsko Etiko. The patients/participants provided their written informed consent to participate in this study.

## Author Contributions

FS and LH designed the research and wrote and edited the manuscript. MJ and MGi provided key antigens and methodology. KL, MI, HG, and SH-G were involved in the larger GRACE observation study, extracted clinical information from the database, and provided clinical samples. FS, MGr, and AE collected the data. FS performed the statistical analysis and visualized the data. LH supervised the experiments and validate the data. All authors contributed to manuscript revision and read and approved the submitted version.

## Conflict of Interest

The authors declare that the research was conducted in the absence of any commercial or financial relationships that could be construed as a potential conflict of interest.

## Publisher’s Note

All claims expressed in this article are solely those of the authors and do not necessarily represent those of their affiliated organizations, or those of the publisher, the editors and the reviewers. Any product that may be evaluated in this article, or claim that may be made by its manufacturer, is not guaranteed or endorsed by the publisher.
