# Supplementary material for: Atypical Antibody Dynamics During Human Coronavirus HKU1 Infections
Source: Front Microbiol. 2022 Apr 27;13:853410. doi: 10.3389/fmicb.2022.853410 (PMC9093712; doi:10.3389/fmicb.2022.853410)
Supplement: Supplementary file 1 [file Data_Sheet_1.PDF]

Supplementary Material

Atypical antibody dynamics during human coronavirus HKU1 infections

Ferdyansyah Sechan[1], Marloes Grobben[1], Arthur Edridge[1], Maarten F. Jebbink[1], Katherine Loens[2,3], Margareta Ieven[3], Herman Goossens[2,3], Susan van Hemert-Glaubitz[4], Marit J. van Gils[1], and Lia van der Hoek[1]

[1] Laboratory of Experimental Virology, Amsterdam Infection and Immunity Institute, Department of Medical Microbiology and Infection Prevention, Amsterdam UMC, University of Amsterdam, The Netherlands
[2] Department of Medical Microbiology, Vaccine & Infectious Disease Institute (VAXINFECTIO), University of Antwerp, Wilrijk, Belgium
[3] Department of Microbiology, University Hospital Antwerp, Edegem, Belgium
[4] Julius Centre for Health Sciences and Primary Care, University Medical Centre Utrecht, Utrecht University, Utrecht, the Netherlands.

*** Correspondence:** Lia van der Hoek (c.m.vanderhoek@amsterdamumc.nl)

This PDF file includes:

- Supplementary Figure S1 – S4
- Supplementary Table S1 – S2

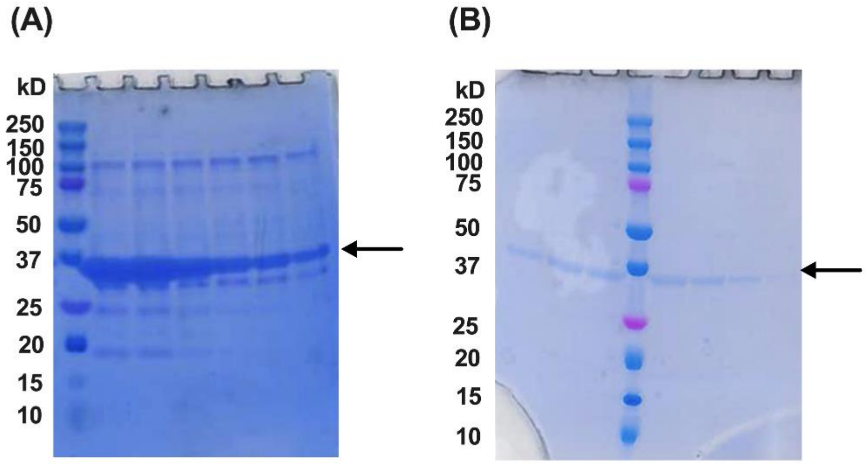

Supplementary Figure S1. Coomassie blue staining on SDS-PAGE gel of purified HKU1-NLCt insoluble fraction after first **(A)** and second **(B)** purification round. HKU1-NLCt antigen is marked with black arrow (33.8 kD). All lanes in the gel picture originates from the same batch of purification.

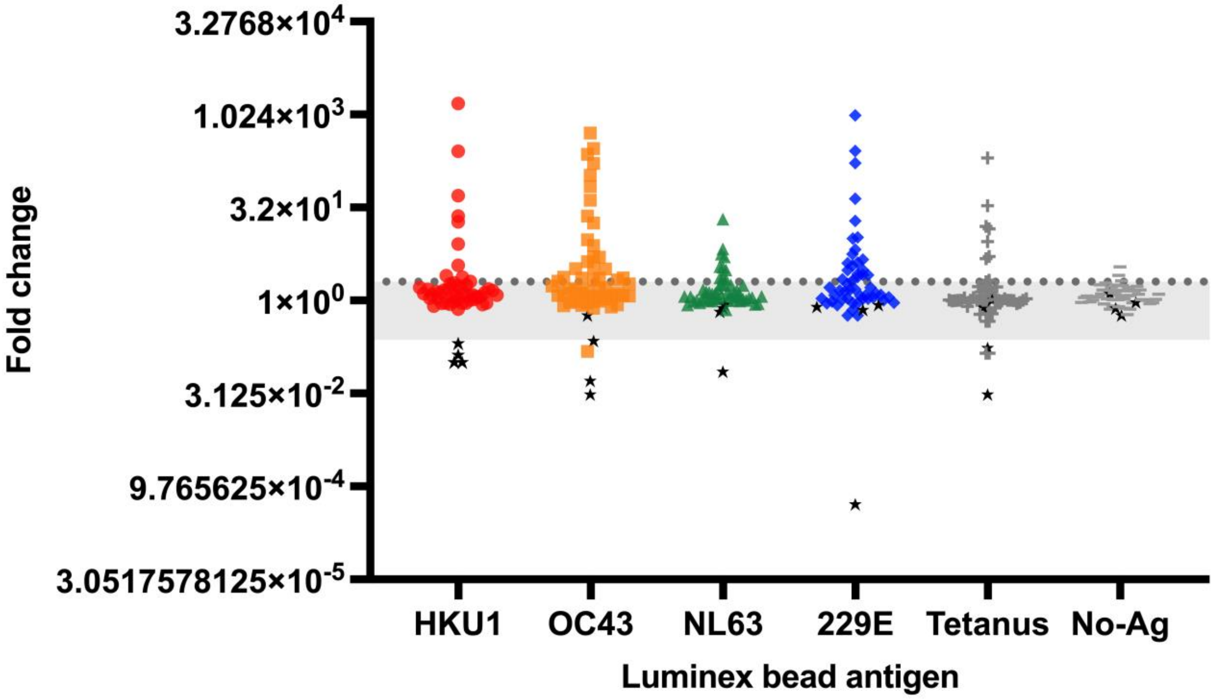

Supplementary Figure S2. The distribution of fold change values from all 54 subjects assayed with Luminex multiplex assay. The antigen used includes the spike of all endemic HCoVs as well as tetanus toxoid as control. Beads without coupled antigen (No-Ag) was included as negative control. The shaded area of the graph represents the Tukey's distribution of the tetanus's fold change values (0.23 to 2.01), by which the upper limit is defined as the cutoff value for significant antibody rise. Black stars represent HCoV-HKU1-infected subjects with low fold change value (subjects HK-01, HK-06, HK-07, and HK-11). One data point of HCoV-OC43 assay below the shaded area (fold change: 0.15) belongs to subject TT-06 (positive for HCoV-229E, matched HCoV fold change of 6.67). Two data points on tetanus assay below shaded area (both fold change: 0.14) belong to subject OC-09 (positive for HCoV-OC43, matched HCoV fold change of 4.23) and subject S0619 (positive for HCoV-HKU1, matched HCoV fold change of 1.39).

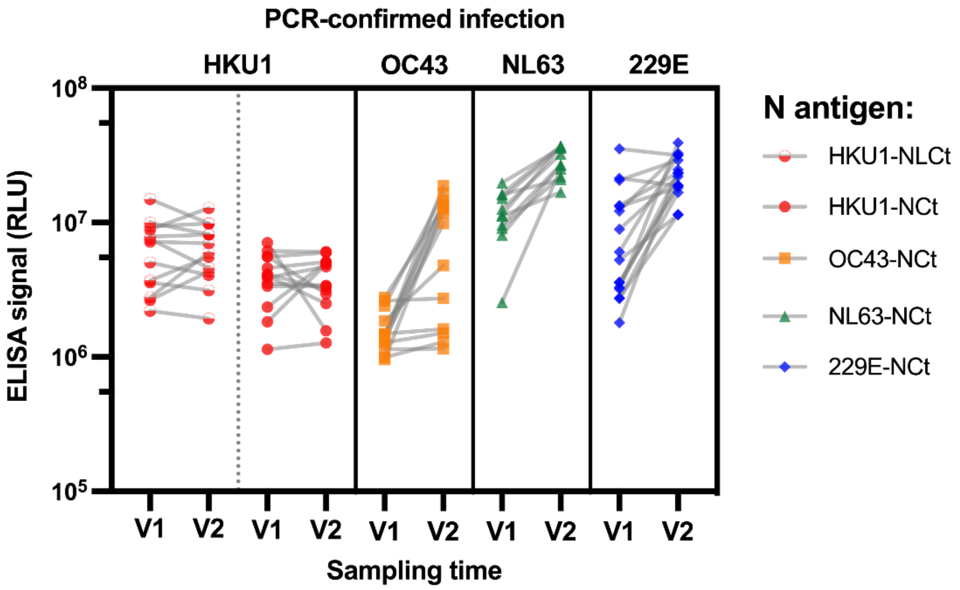

Supplementary Figure S3. The ELISA signal values (RLU) of serum samples from 54 HCoV-infected subjects assayed by matched N antigens. V1 and V2 refer to sampling time at the start or study enrollment and at follow up (between 3-5 weeks), respectively. For HCoV-HKU1-positive subjects, both HKU1-NLCt (half-filled red circles) and HKU1-NCt (full red circles) were used for the N assay. Grey lines represent the V1 and V2 values from one subject. NCt ELISA raw RLU values for all endemic coronaviruses were obtained from Edridge et al. (2020).

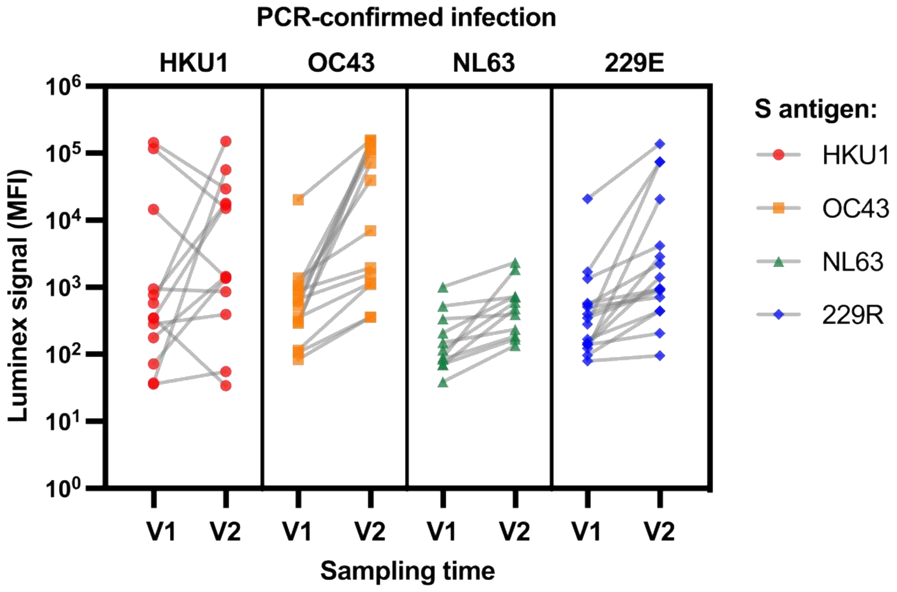

Supplementary Figure S4. The Luminex signal value (MFI) of serum samples from 54 HCoV-infected subjects assayed by matched S antigens. V1 and V2 refer to sampling time at the start or study enrollment and at follow up (between 3-5 weeks), respectively. Grey lines represent the V1 and V2 values from one subject.

Supplementary Table S1. Serum antibody level of HCoV-HKU1-infected subjects assayed by spike Luminex and nucleocapsid ELISA.

ID	HKU1 spike (MFI)		HKU1 NLCt (RLU)		HKU1 NCt (RLU)		Fold change spike	Fold change NLCt	Fold change NCt
	V1	V2	V1	V2	V1	V2			
HK-01	117617	15017	14915000	9828000	5604000	2952000	0.13	0.66	0.53
HK-02	942.5	861.5	7877000	8138000	5551000	5955000	0.91	1.03	1.07
HK-03	344	17053	2770000	5994500	1832000	4898000	**49.57**	**2.16**	**2.67**
HK-04	575	150401.5	9363000	9857000	6126000	6077000	**261.57**	1.05	0.99
HK-05	36	55	7208000	6997000	3533000	3396000	1.53	0.97	0.96
HK-06	348	34	9952500	8158500	4142000	3173000	0.1	0.82	0.77
HK-07	14497	1392	2204000	1934500	3353000	3365000	0.1	0.88	1
HK-08	72	1355	3592000	3128500	1138000	1272000	**18.82**	0.87	1.12
HK-09	284	394	5031000	4068000	7088000	1573000	1.39	0.81	0.22
HK-10	37	56774	3701000	5571000	3895000	4693000	**1534.43**	**1.51**	1.2
HK-11	144764	29512.5	7528000	4576000	4011000	2495000	0.2	0.61	0.62
HK-12	176	1447	8859000	12785000	4581000	5086000	**8.22**	**1.44**	1.11
HK-13	772	17895	2659000	4321000	2347000	5055000	**23.18**	**1.63**	**2.15**

Bold values: fold change values above cutoff of significant antibody increase (2.01 for spike assay, 1.40 for nucleocapsid assay)

Underlined values: fold change value below cutoff of significant antibody decrease (0.23 and 0.70 for spike and nucleocapsid assay)

Supplementary Table S2. Serum antibody level of HCoV-infected subjects assayed by matched spike Luminex and nucleocapsid ELISA.

ID	Matched spike (MFI)		Matched NCt (RLU)		Fold change spike	Fold change NCt
	V1	V2	V1	V2		
OC-01	1059	112775.5	953700	12630000	**106.49**	**13.24**
OC-02	307	71621	1456000	14540000	**233.29**	**9.99**
OC-03	106.5	362	1864000	4807000	**3.40**	**2.58**
OC-04	622.5	103170	1316000	11750000	**165.73**	**8.93**
OC-05	935	39293.5	2624000	2718000	**42.03**	1.04
OC-06	664.5	1577	1396000	9867000	**2.37**	**7.07**
OC-07	1373	7000	1278000	1505000	**5.10**	1.18
OC-08	526.5	151733	1474000	13550000	**288.19**	**9.19**
OC-09	84	355.5	991000	1292000	**4.23**	1.30
OC-10	20152	156767	1458000	18790000	**7.78**	**12.89**
OC-11	115.5	1106	1135000	1153000	**9.58**	1.02
OC-12	351	1148	2772000	13060000	**3.27**	**4.71**
OC-13	290	149183	1494000	1613000	**514.42**	1.08
OC-14	904	1953	2398000	16550000	**2.16**	**6.90**
NL-01	523.5	721	11190000	16900000	1.38	**1.51**
NL-02	1010	2344	16110000	20970000	**2.32**	**1.41**
NL-03	117	594	8094000	26710000	**5.08**	**3.3**
NL-04	89	1842.5	2560000	25160000	**20.7**	**9.83**
NL-05	70	477	11380000	36860000	**6.81**	**3.24**
NL-06	149	234.5	19820000	36000000	1.57	**1.82**
NL-07	71	168.5	15240000	36220000	**2.37**	**2.38**
NL-08	336	392	9573000	26990000	1.17	**2.82**
NL-09	208.5	737	12630000	32370000	**3.53**	**2.56**
NL-10	85	186	16340000	37370000	**2.19**	**2.29**
NL-11	39	135.5	9115000	22060000	**3.47**	**2.42**
TT-01	140	1411	1795000	24690000	**10.08**	**13.75**
TT-02	123	20575	3566000	18750000	**167.28**	**5.26**
TT-03	1686.5	74562	20650000	29070000	**44.21**	**1.81**
TT-04	281	73585	3218000	39170000	**261.87**	**12.17**
TT-05	545	2223	6035000	31550000	**4.08**	**5.23**
TT-06	20785.5	138652.50	12240000	32750000	**6.67**	**2.68**
TT-07	347	896	3604000	18930000	**2.58**	**5.25**
TT-08	397	952	35270000	31720000	**2.4**	0.9
TT-09	147.5	2869	3303000	23210000	**19.45**	**7.03**
TT-10	97	443	2728000	16810000	**4.57**	**6.16**
TT-11	1344	4169.5	21300000	18770000	**3.1**	0.88
TT-12	138	205	2740000	11490000	1.49	**4.19**
TT-13	580	938	13390000	23640000	1.62	**1.77**
TT-14	509	713	8897000	22140000	1.4	**2.49**
TT-15	79	96	13180000	18230000	1.22	1.38
TT-16	168	440	5256000	11410000	**2.62**	**2.17**

Bold values: fold change values above cutoff of significant antibody increase (2.01 for spike assay, 1.40 for nucleocapsid assay)
